# Cell grazing and *Haemonchus contortus* control in sheep: lessons from a two-year study in temperate Western Europe

**DOI:** 10.1038/s41598-019-49034-y

**Published:** 2019-09-03

**Authors:** C. Ruiz-Huidobro, L. Sagot, S. Lugagne, Y. Huang, M. Milhes, L. Bordes, F. Prévot, C. Grisez, D. Gautier, C. Valadier, M. Sautier, P. Jacquiet

**Affiliations:** 1 UMR INRA/ENVT 1225 IHAP, UMT Santé des Petits Ruminants, Ecole Nationale Vétérinaire de Toulouse, BP 87614, 31 076 Toulouse, cedex 03 France; 2 CIIRPO, ferme du Mourier, 87800 Saint-Priest, Ligoure France; 3 GenPhySE, Université de Toulouse, INRA, INPT, ENVT, Castanet-Tolosan, France

**Keywords:** Parasite development, Parasite biology

## Abstract

Managing infections of sheep with anthelmintic resistant gastrointestinal nematodes (GIN) is a major challenge for sheep producers in Western Europe. New methods of grazing management have been poorly explored as a component of an integrated and sustainable control of these parasites. Therefore, the purpose of this study was to evaluate the effect of two different types of grazing systems of sheep (intensive cell grazing *versus* conventional rotational grazing) on GIN infections over two years in a farm located in a temperate environment of Western France. When considering the whole study, the type of grazing system did not influence significantly the intensity of egg excretions of adult ewes even if the proportion of ewes excreting high numbers of GIN eggs was higher in cell grazing system than in rotational grazing system. The most striking result of this survey was the effect of grazing system on the GIN species composition harbored by ewes and by their lambs: with time, the proportions of *H*. *contortus* infections were lower in cell grazing system than in rotational grazing system. In conclusion, the cell grazing system, as implemented in this study, could limit the importance of this highly pathogenic nematode species in sheep.

## Introduction

Gastrointestinal nematode (GIN) infection is one of the major health issues in sheep worldwide. In temperate countries of Western Europe, three GIN species dominate the digestive helminthofauna of sheep: *Teladorsagia circumcincta*, *Haemonchus contortus* and *Trichostrongylus colubriformis*. All of them are responsible for growth retardation and milk production losses^1^; however, only *H*. *contortus* infections can lead to high mortality rates in lambs and ewes, especially when climatic conditions are optimal for the development of free-living stages on pastures and when sheep are exposed to them at grazing. For decades, the control of GIN relied exclusively on anthelmintic treatments. However, the effectiveness of anthelmintics, and the welfare and production benefits they bring, is threatened by the increasing prevalence and severity of anthelmintic resistance. Western Europe is more and more facing multiple-resistance in GIN^2–4^. In Southwestern France, the first ivermectin and benzimidazoles resistant *Haemonchus contortus* isolate has been reported recently^5^. In this context, control strategies for GIN that minimize the use of anthelmintics are of increasing importance not only in organic farms but also in conventional farms. Many components of an integrated control program have been investigated such as selection of a genetic resistance in sheep^6^, vaccination against *H*. *contortus* infection^7^ or the use of condensed tannins in alimentation^8^. Some other strategies are focusing on the limitation of pastures contamination by using nematofagous fungi^9^. In comparison, the effects of pastures management on GIN infections in sheep have received less attention with the notable exception of mixed or alternate sheep and cattle grazing^10^ or studies performed in Australia (Cicerone project in New South Wales), where the effects of cell grazing system on GIN infections were evaluated in comparison with a typical rotational grazing system^11,12^. Cell grazing system is characterized by the division of pastures in many small “cells” (less than 0.5 hectares (ha)) in which sheep or cattle are allowed to graze for a very short time (usually less than two days) at a very high instant stocking rate (about 500 sheep per hectare). This is strictly different from typical rotational grazing system where grazing periods are longer (one week or more) and instant stocking rates four or five times less important than in cell grazing system. In addition, the mean rest periods for a cell are commonly longer than the mean rest periods in rotational grazing system. All these factors have probably many effects on the development and the survival of free living stages of GIN on pastures. Indeed, in the Australian studies, cell grazing system was able to markedly reduce fecal egg counts in lambs and ewes during a six-year long survey. According to these authors, cell grazing system may be an effective way to control *H*. *contortus* infections in temperate environments. Even if cell grazing system in sheep farming is extending in Western France, no specific survey has been performed to evaluate the effect of this new pasture management on GIN epidemiology for such agroclimatic conditions. We do believe that findings obtained in an Australian context could not be fully extrapolated into a Western European context and that specific studies are necessary.

Therefore, the aims of this study were i) to compare the intensities of GIN egg excretions during a 2-year period between cell grazing system and typical rotational grazing system and ii) to evaluate a potential effect of cell grazing system on frequencies and intensities of *H*. *contortus* infections. This experiment was conducted in a commercial farm from Western France. According to the Australian study, cell grazing system seems to have a deep impact on *H*. *contortus* populations; however, the benefit of cell grazing system in terms of intensities of GIN egg excretions was not observed in the present study.

## Results

**Figure 1:**
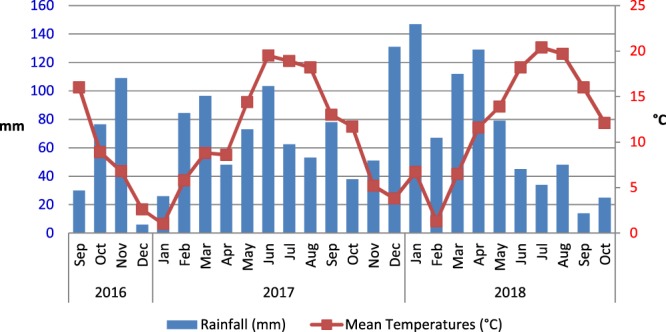
Monthly rainfall (blue bars) and monthly average temperatures (red line) at Le Mourier farm during the experimental period (September 2016 to October 2018).

Monthly rainfalls and monthly average temperatures, recorded in the farm during the study period, are presented in Fig. 1. The annual rainfalls in 2016 and 2017 were respectively 944 mm and 845 mm which were slightly above the decennial mean of rainfalls in this location (816 mm). The total rainfall from January to October 2018 was 700 mm which was very close to the sum of rainfalls from January to October 2017 (663 mm) and the decennial mean of rainfall during the ten first months of the year (668 mm). Therefore, the total rainfalls recorded during the study were within the range of expected values. However, the repartitions of rains differ from one year to another. The rainfalls were relatively homogenous in 2017 but were concentrated in winter and spring in 2018 and followed by a deficit from June to October 2018. Mean temperatures observed during the study were within the range usually recorded in this region.

**Figure 2:**
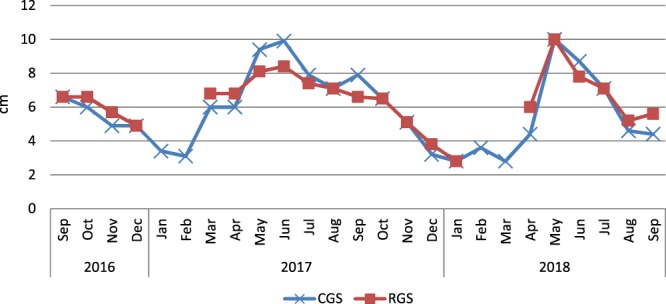
Seasonal patterns of average grass heights (in cm)  according to the grazing system. CGS = cell grazing system; RGS = rotational grazing system.

Mean graze periods and mean rest periods by year and management system, recorded in Le Mourier farm, are presented in Table 1. According to the principles of cell grazing, the mean graze periods in cell grazing system did not exceed 3 days with a mean of 0.5 day in 2016 and around one day in 2017 and 2018. By contrast, these mean graze periods were more important in rotational grazing system (6 days in 2016, 2017 and 2018). Mean rest periods for a cell were stable in cell grazing system (between 45 and 47 days) during the study; however, these periods fluctuated more in rotational grazing system: long in 2016 (44.6 days), they diminished in 2017 (38 days) and in 2018 (35.3 days). The difference between mean rest periods of cell grazing and rotational grazing systems was not significant in 2016 but was significant (p < 0.05) in 2017 and 2018. As expected in this temperate environment of Western Europe, the grass heights varied greatly from one season to another (Fig. 2). Similar seasonal patterns were observed in both systems; however, ewes in cell grazing system were grazing during winter (excepted during the month after lambing - March) whereas ewes in rotational grazing system were indoors from January to end of March. In consequence, during the whole study, ewes in cell grazing system grazed six months when grass height was below 4 cm (January, February and December 2017, January, February, March 2018) while ewes in rotational grazing systems grazed only two months (December 2017 and January 2018) in these conditions.

**Table 1:** Mean graze period and mean rest period by plot, by year and by management system.

	Cell grazing system			Rotational grazing system		
	2016	2017	2018	2016	2017	2018
Mean graze period (days)	0.5	1.1	1	6	6	6.6
Mean rest period (days)	45.4	45.4	47.2	44.6	38	35.3
Time of housing	End of February				Mid-January	
Time of turnout		End of March			End of March

**Figure 3:**
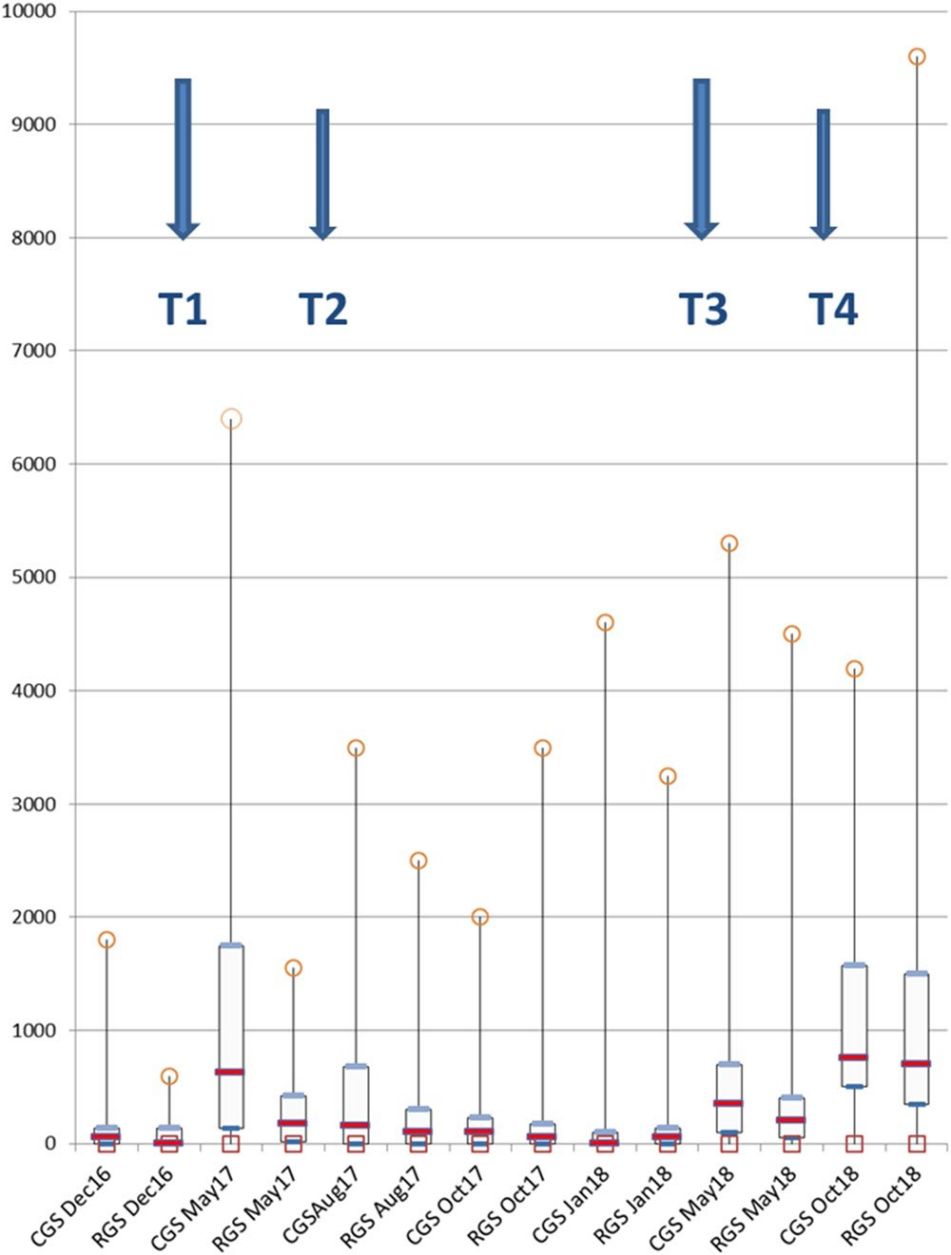
Intensities of GIN egg excretions in EPG over the study period according to the grazing system (CGS = cell grazing system and RGS = rotational grazing system). Minimal (squares) and maximal (circles) values of EPG (egg per gram), median values (red horizontal bars), Q1 and Q3 (blue horizontal bars). T1 and T3: systematic treatments with netobimin (HAPADEX, 20 mg/kg BW), T2: treatment with levamisole (BIAMINTHIC 5%, 7.5 mg/kg BW), T4: treatment with oral moxidectin (CYDECTINE ORALE, 0.2 mg/kg BW).

Seasonal patterns of GIN egg excretions in ewes are presented in Fig. 3. A very high individual variability was noted whatever the date of sampling in both grazing systems. Very low eggs per gram (EPG) values were recorded in both grazing systems in December 2016, October 2017 and January 2018 apart from some animals. Ewes in cell grazing system excreted more eggs than ewes in rotational grazing system in May, August 2017 and May 2018. However, the type of grazing system did not influence significantly the intensity of egg excretions when considering the whole study (Table 2). Nevertheless, the proportion of ewes excreting more than 500 EPG was significantly higher in cell grazing system (34%) than in rotational grazing system (23.3%) (Chi-square = 6.19; p < 0.05). A similar trend was observed for ewes excreted more than 1000 EPG (Table 3) but the difference was not significant. A strong effect of season on EPG was shown (Table 2). In lambs, intensities of GIN egg excretions differed between systems (Table 4). In both years (2017 and 2018), lambs born in rotational grazing systems excreted significantly more eggs than lambs born in cell grazing system (p = 0.018 and p = 0.002 respectively).

**Table 2:** The effects of grazing system (cell grazing system vs Rotational grazing system), season, age of ewes and interactions between factors on eggs per gram (EPG), body condition score (BCS) and packed cell volumes (PCV) (data from the whole study are considered and analyzed).

Variable	Cell grazing system mean values, (+/− SD) *median*	Rotational grazing system mean values, (+/− SD) *median*	*p-value*				
			Grazing system	Season	Age of ewes	System-season	System-age
EPG	575 (+/− 932) 150	469 (+/− 1080) 100	*0*.*316*	<***0***.***001***	*0*.*093*	***0***.***028***	*0*.*990*
PCV	29.2 (+/− 3.8) 29	29.9 (+/− 3.8) 30	*0*.*220*	<***0***.***001***	***0***.***013***	***0***.***007***	*0*.*352*
BCS	2.34 (+/− 0.56) *2*.*5*	2.62 (+/− 0.58) *2*.*5*	<***0***.***001***	<***0***.***001***	***0***.***02***	<***0***.***001***	*0*.*067*

**Table 3:** Percentages of ewes showing GIN egg excretion according to the date of sampling and the grazing system.

Date	Cell grazing system				Rotational grazing system			
	Total number of ewes measured	Positive egg excretion (%)	Over 500 EPG (%)	Over 1000 EPG (%)	Total number of ewes measured	Positive egg excretion (%)	Over 500 EPG (%)	Over 1000 EPG (%)
Dec16	30	56.7	6.7	3.3	30	46.7	3.3	0
May17	28	89.3	57.1	39.3	26	73.1	23.1	15.4
Aug17	24	62.5	29.2	20.8	25	60	16	8
Oct17	35	71.4	14.3	8.6	36	55.6	16.7	5.6
Jan18	34	47.1	14.7	5.9	34	61.8	14.7	8.8
May18	41	95.1	39	14.6	40	77.5	22.5	12.5
Oct18	35	97.1	77.1	34.3	33	97	66.7	39.4
**Whole study**	**227**	**74**.**2**	34*	**18**.**1**	**224**	**67**.**4**	***23***.3*	**12**.**8**

Percentages of ewes showing high egg excretions (over 500 EPG and over 1000 EPG) in both grazing systems. * indicates that the difference is significant.

**Table 4:** Intensities of GIN egg excretions (mean, standard deviation and median) in 25 lambs per grazing system in June 2017 and in June 2018 (just before weaning, 100 days old lambs).

	Cell grazing system (N = 25 lambs per year)			Rotational grazing system (N = 25 lambs per year)		
	Mean	SD	Median	Mean	SD	Median
June 2017	208*	375	100	346*	341	250
June 2018	70*	114	15	370*	635	150

*indicates that the difference between EPG of lambs from cell grazing system and rotational grazing system was significant.

Body condition scores (BCS) were lower in cell grazing system ewes than in rotational grazing system ewes during the whole study (p < 0.001) with the exception of October 2018 where BCS were significantly higher in ewes from the cell grazing system (Table 5). BCS failed to low values after lambing and improved again in autumn till the next lambing period (Table 5).

**Table 5:** Body condition scores (BCS) and packed cell volumes (PCV) in ewes according to the date of sampling and the grazing system.

Date	Cell grazing system			Rotational grazing system		
	Total number of ewes measured	Mean BCS (SD)	Mean PCV (SD)	Total number of ewes measured	Mean BCS (+/− SD)	Mean PCV (+/− SD)
Dec16	30	2.58 (0.4)	30.5 (3.1)	30	3.18 (0.33)	31.9 (2.9)
May17	28	2 (0.45)	26.3 (3.4)	26	2.6 (0.64)	27 (2.9)
Aug17	24	2.18 (0.52)	26.4 (2.75)	25	2.47 (0.62)	27.5 (3.25)
Oct17	35	2.44 (0.62)	29.3 (3.2)	36	2.61 (0.57)	29.2 (3.1)
Jan18	34	2.66 (0.47)	32.3 (3.6)	34	2.93 (0.43)	34.2 (2.7)
May18	41	1.9 (0.36)	28 (2.8)	40	2.27 (0.48)	29.3 (3)
Oct18	35	2.55 (0.56)	30.2 (4)	33	2.4 (0.4)	29.1 (3.5)
**Whole study**	**227**	**2**.**34 (0**.**48)**	**29**.**2 (3**.**3)**	**224**	**2**.**62 (0**.**49)**	**29**.**9 (3**.**05)**

Mean values and standard deviations (SD).

Packed cell volumes (PCV) showed similar pattern in both grazing systems and the type of grazing did not influence this parameter (Table 2). EPG, BCS and PCV were significantly correlated (r = 0.404 between PCV and BCS values; r = −0.296 between PCV and EPG values and r = −0.347 between BCS and EPG values).

**Figure 4:**
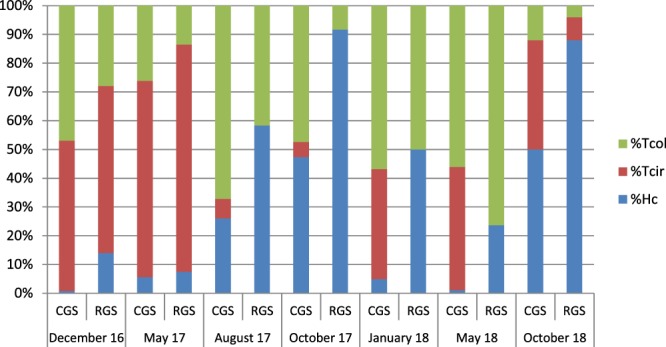
Species composition (%) of infective larvae obtained from fecal samples of ewes conducted in CGS (cell grazing system) or RGS (rotational grazing system) during the study. Tcol = *Trichostrongylus colubriformis*; Tcir = *Teladorsagia circumcincta* and Hc = *Haemonchus contortus*).

Figure 4 shows the species composition of infective larvae obtained from fecal samples of ewes conducted in cell grazing system and in rotational grazing system evaluated by Real-Time PCR. As no clear differences in species composition appear between ages in a same grazing system, results of different ages of ewes within a category of grazing system have been pooled together. In the beginning of the survey (December 2016 and May 2017), the helminthofauna of both grazing systems appeared similar with *Teladorsagia circumcincta* as the dominant species. From August 2017 to October 2017, the species composition changed sharply with the decreased proportion of *T*. *circumcincta* and a larger proportion of *H*. *contortus*, especially in rotational grazing system. From January 2018 to October 2018, helminthofauna showed divergence between the two grazing systems. *T*. *circumcincta* was well represented in cell grazing system while this species was very rare in rotational grazing system. The most striking finding of this survey is that *H*. *contortus* seems to be in minority in cell grazing system in January and May 2018. By contrast this species was well represented in rotational grazing system ewes. Finally, in October 2018, *H*. *contortus* was present in cell grazing system (50%) but in lower proportion than in rotational grazing system (88%). Altogether, these results seem to indicate that the proportion of *H*. *contortus* in larval cultures of ewes conducted in cell grazing system was lower than in larval cultures of ewes in rotational grazing system. This trend was not observed in the beginning of the study but in the second half on the study period. Morphological and molecular identifications lead to the same conclusions (data not shown).

**Figure 5:**
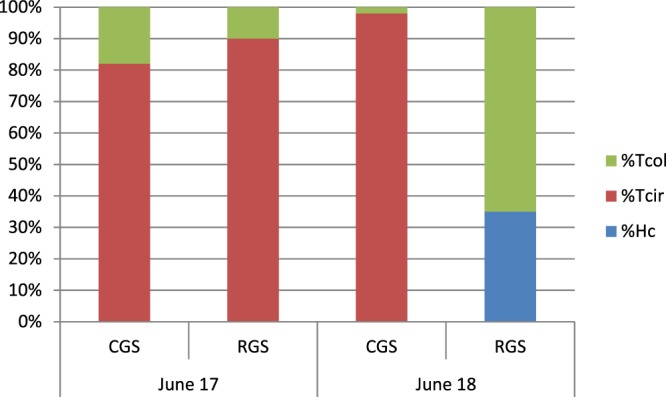
Species composition (%) of infective larvae obtained from fecal samples of lambs conducted with their mothers in CGS (cell grazing system) or RGS (rotational grazing system) in June 2017 (25 lambs per grazing system) and June 2018 (25 lambs per grazing system). Tcol = *Trichostrongylus colubriformis*; Tcir = *Teladorsagia circumcincta* and Hc = *Haemonchus contortus*).

Regarding the species composition assessed by Real-Time PCR, helminthofauna of lambs were quite similar in 2017 in cell grazing system and rotational grazing system (with the predominance of *T*. *circumcincta*) but differed a lot in 2018 with a majority of *T*. *circumcincta* in lambs from the cell grazing system and mixed *H*. *contortus* and *T*. *colubriformis* infections in lambs from the rotational grazing system (Fig. 5). Again, morphological and molecular identifications of infective larvae obtained from lambs were highly concordant.

## Discussion

The two main findings of this survey were that i) the effect of the type of grazing system on the intensity of egg excretions was not significant and that ii) the species composition was affected by the type of grazing system in the second half of the survey.

In the Australian survey^11,12^, cell grazing system clearly improved worm control relative to rotational grazing system as evidenced by both lower overall GIN eggs excretions and fewer anthelmintic treatments required. Furthermore, acute-haemonchosis was recorded in rotational grazing system but not in cell grazing system in the Australian context. These findings have not been recovered in the present study. On the contrary, the proportion of ewes excreting high numbers of GIN eggs was superior in cell grazing system than in rotational grazing system during the whole study. Moreover, the intensities of egg excretions were higher in cell grazing system than in rotational grazing system at three sampling dates (May and August 2017; May 2018). However, it is unclear if this is due to the grazing system only or to a combination of grazing system and individual variations of susceptibility to GIN infections in the studied ewes. Nevertheless, the effect of grazing system was not significant when the entire period of study was taken into account. How can we explain this difference between both studies? The grazing periods per cell in cell grazing system were similar in French and Australian studies (between 0.5 and 1 day) suggesting that these short periods would have largely prevented autoinfection from the current grazing cycle in both sites. However, the rest periods of “cells” were twice longer in the Australian study than in the French study (a mean of 98 and 45 days respectively, and rarely below 50 days and sometimes over 150 days in the Australian case). Such long rest periods usually allow time for significant larval die-off in most cases. Livestock and grazing in the temperate climate of Western France claimed for a rapid come back of animals to previously grazed plots as longer rest periods would lead to poor forage quality of grass. Therefore, the rest periods in the cell grazing system performed in “Le Mourier” farm were probably too short to ensure a substantial higher larval die-off in comparison to rotational grazing system. Another explanation may come from the difference of total grazing duration during one year: 11 months in the cell grazing system compared to 9–10 months in the rotational grazing system, this difference allowing a longer period of GIN infections in first grazing system. Grass height is also an important factor in the probability of larval intake by sheep in pastures. Below 4 cm, this probability could increase as many GIN infective larvae are concentrated between the soil level and 4 cm height^13^. During the present study, ewes from cell grazing system grazed 6 months when grass height was below 4 cm compared to only two months in rotational grazing system. Altogether, these three factors may explain why no substantial benefit of cell grazing system in the intensities of GIN egg excretions was observed in the present experiment. Nevertheless, this conclusion cannot be applied to lambs as slightly but significant higher egg excretions were recorded in rotational grazing system compared to cell grazing system. The reasons of this difference are not yet clear. In both years, lambs had the same age and grazed for a similar cumulative time when sampled in both grazing systems. One can notice that grass height was higher in cell grazing system than in rotational grazing system in May and June 2017, rending probably GIN infective larvae less available for lambs in cell grazing system, but this factor could not act in 2018 as the difference of grass height did not occur this year.

In the Australian study, the efficacy of cell grazing system in worm control was associated with a shift in the relative abundance of the different parasite genera. As the experiment progressed, there was a reduction in the proportion of *H*. *contortus* in the larval cultures of animals conducted in cell grazing system with a concomitant increase in the proportion of *T*. *circumcincta*^12^. According to these authors, the lower proportion of *Haemonchus* infections seen in cell grazing system reflected probably its greater susceptibility to lifecycle disruption by grazing management. Similar shift in species composition has been observed in ewes of the present study. Rapid development from eggs to L3 (three to four days) of *H*. *contortus* and *Trichostrongylus* spp. has been largely reported under optimal conditions (25 °C and moisture available in the first week after eggs’ deposition) both in laboratory and in the field^14^. In cell grazing system, little and probably no autoinfection would occur in sheep during a given short grazing period: therefore, L3 need to survive until the next grazing period to infect a host. On the contrary, *H*. *contortus* and *Trichostrongylus* spp. autoinfections are likely to occur in rotational grazing system because of the longer grazing period (one week or more) on a given plot, at least during the summer time when temperatures are suitable for rapid development. The different GIN genera show high variations in their ability to develop in cold temperatures: eggs of the *T*. *circumcincta* species are able to develop into L3 at 5 °C; this is very unlikely to occur with *H*. *contortus*^15^. Therefore, when CGS ewes are grazing during winter, they probably ingest a more important proportion of *T*. *circumcincta* L3. In spring, already established *T*. *circumcincta* adult worms could have a negative impact on the establishment and the development of newly ingested *H*. *contortus* L3^16^. In the present study, ewes in rotational grazing system were kept indoors during winter and some *T*. *circumcincta* infections were probably avoided by this way. The capacity of the L3 to survive desiccation conditions appears to be dependent on the GIN species. Of the three GIN species examined in the study of Chylinski *et al*.^17^, *H*. *contortus* had the lowest capacity to survive desiccation, followed by *T*. *colubriformis* and the *T*. *circumcincta*. It means that *H*. *contortus* species could be more affected than other species by long rest periods of the plots. Even if the difference of rest period durations was relatively moderate in the present study, it could be a challenge for *H*. *contortus* L3 to survive 10 or 15 days more in cell grazing system than in rotational grazing system before the comeback of hosts in a given plot, especially during summer periods. Taken together, these data suggest that cell grazing system could affect more preferentially the opportunities of *H*. *contortus* infections in grazing sheep than those of *T*. *circumcincta*. Species composition in lambs seems to reflect perfectly the species composition found in ewes with a common dominance of *T*. *circumcincta* in cell grazing system and rotational grazing system during spring 2017 and a high discrepancy in spring 2018, dominance of *T*. *circumcincta* in cell grazing system lambs and of *H*. *contortus* and *Trichostrongylus* spp. in rotational grazing system. This similar pattern in the helminthofauna in ewes and lambs probably confirmed the fact that eggs deposited by ewes in early spring were the main source of contamination of their offspring. In the present study, lambs could be considered as tracers lambs: they are revealing the current composition of helminthofauna on pastures elicited by adult ewes. Data of species composition obtained in lambs strongly support those obtained in ewes: the relative proportion of *H*. *contortus* larvae in the larval cultures of both lambs and ewes seems to be lower in cell grazing system than in rotational grazing system. Body condition scores were significantly lower in cell grazing system than in rotational grazing system when considering the whole study with one exception in October 2018. This could be due to several factors: ewes in rotational grazing system received complement diet during three months when they remained indoors in winter. In the same time, ewes in cell grazing system were grazing at a moment when height grass was low and of probably poor quality. Ewes in cell grazing system were indoors for a shorter period (one month) and received less complement diet than ewes from the other grazing system and this was probably not sufficient to fill the gap. Climatic reasons could also act to favor poor quality of forage during winter.

## Conclusions

We conclude that cell grazing system, as conducted in this study, is not able to decrease the intensities of GIN eggs excretions in grazing ewes compared to rotational grazing system in temperate conditions of Western France contrary to what was observed in Australia. However, cell grazing system could provide an additional tool to limit the *H*. *contortus* infections, which represent today an important issue for sheep farm sustainability, regarding the increased number of reports of multiple-resistance to anthelmintics in this species. Nevertheless, there are still great challenges with implementation of cell grazing system and it is worth noticed that such system was applied primarily to improve animal production and the sustainability of grazing systems rather than to limit the effects of GIN. Further studies on impact of cell grazing system in other ecological and management conditions are required in Southwestern France before recommending this grazing strategy to limit *H*. *contortus* infections in sheep.

## Materials and Methods

This study was conducted under the agreement N° 2015260-002-ddcspp (Direction Départementale de la Cohésion Sociale et de la Protection des Populations de la Haute Vienne).

### Study site

The study took place on a commercial meat sheep farm, called “Le Mourier”, located 25 km south of Limoges (Nouvelle Aquitaine region, France). Le Mourier farm (320 m altitude, 45°6622 North; 1°2925 East) has a temperate climate with the majority of rainfall in winter and spring. The average annual rainfall is ~900 mm. Summers days are usually dry and hot, sometimes very hot (up to 35 °C), whereas winters are cold (mean temperatures between 0 and 5 °C in December, January and February).

### Experimental design

This experiment was a longitudinal study of GIN infections under two different farm management systems over a 2-year period from September 2016 to October 2018. In this study, sheep were “Vendéenne” sheep, a meat sheep breed from Western France. The classes of sheep monitored during the study were ewes (females over 2 years of age) and their lambs (<4 months of age). 300 Vendéenne ewes have been divided into two groups of 150 individuals, one group conducted on a rotational grazing system and the other group on a cell grazing system. In the farm, 15 ha were dedicated to each grazing system i.e, the stocking rate was similar in both systems, 10 ewes/ha. These 15 ha were divided into 10 plots in cell grazing system and 11 plots in rotational grazing system but each plot in cell grazing system was subdivided into small “cells” of 0.20 ha each. Regarding the rotational grazing system, a housing period of 60 days minimum was strictly respected from January to March, while in cell grazing system, only a one-month period of housing was allowed mainly to avoid predation of young lambs by foxes. Lambing period extended from mid-February to mid-March for the two grazing systems. In both systems, lambs were allowed to graze with their mothers from one-month of age and are weaned at 120 days. Turn out occurred in rotational grazing system between end of March and mid-April when cumulative degree-days were over 300° days. For one plot, mean duration of grazing period was six days in rotational grazing system (ranged from 1 to 19 days) whereas this mean duration was only one day for a cell in cell grazing system (ranged from 0.1 to 3.3 days) due to the division of one plot in several “cells”. According to the experience of the previous years, the expected mean rest period for a plot was 21 days minimum in rotational grazing system and between 20 and 60 days for a cell in cell grazing system. Anthelmintic treatments were decided by the farm manager and followed a drenching program previously established by the local veterinarian. All 300 ewes of this study have been treated with doramectine and triclabendazole in September 2016 at the beginning of the survey. Netobimin treatment was applied to control *Dicrocoelium dendriticum* infections on February 2017 and in January 2018 i.e. to prepare lambing. Another anthelminthic treatment was done in May 2017 (levamisole) and in June 2018 (oral moxidectin). All around the study, ewes from the cell and from the rotational grazing systems have been treated in the same time with the same molecules.

### Measurements

Rainfall and temperatures data were accessed from a meteorology station located within the farm. Mean graze periods and mean rest periods were calculated plot by plot (rotational grazing system) or cell by cell (cell grazing system) thanks to data recorded by the personnel of Le Mourier farm. Height of grass was evaluated by the same person during the whole study, plot by plot (both systems), by using a special equipment (JENQUIP). 30 measures were done by hectare and the mean value was reported in the data sheet. These measures were done monthly during the whole period of study on a limited set of plots (three per grazing system).

Between 25 and 40 individual fecal samples were collected per rectum in ewes at seven sampling dates (December 2016, May, August and October 2017 and January, May and October 2018). These ewes were always divided into different age classes: ewes born in 2012, 2013 and 2014 from December 2016 to May 2017 dates of sampling; ewes born in 2012, 2013, 2014, 2014 and 2015 from October 2017 to January 2018 sampling sessions and ewes born in 2012, 2013, 2014, 2015 and 2016 from May to October 2018. These modifications were necessary, because some ewes present at the beginning of the survey have been culled during the study for independent reasons of the parasitological survey (abortion, failure of mating, foot’s aches…). In addition, 50 blood and fecal samples of lambs (25 from the cell grazing system and 25 from the rotational grazing system) were collected in June 2017 and in June 2018 in order to measure the intensities of GIN egg excretions and their packed cell volumes. Fecal samples collected in ewes and lambs were sent in a cold box (+4 °C) by high-speed (less than 24 hours of travel) postal service (CHRONOPOST) to the National Veterinary School of Toulouse. In the laboratory, the intensity of nematode egg excretion in 3 g of each individual sample was estimated using the modified McMaster technique^18^ within 48 hours after the sampling. During the whole study, a total of 227 and 224 fecal egg counts have been done in cell grazing system and in rotational grazing system respectively. The remaining feces were pooled by grazing system (rotational versus cell grazing systems) and by age category (ewes of different ages and lambs). Three to five grams per individual were pooled in a plastic box, cultured for 12 days at 24 °C+/−1 °C and humidified with tap water every two days. Then, third stage larvae (L3s) were recovered by filling each beaker with water (25 °C) and inverting it on to a Petridish^19^. L3s were first suspended in a total volume of 40–45 mL tap water before centrifugation (4500 rpm during 10 minutes) in order to concentrate them into a 5 mL final volume. Thereafter, L3s were stored at +4 °C until counting and identification. Larval identification was performed by using: (1) the morphological criteria of Van Wyk and Mayhew^20^ and (2) a Real-Time PCR analysis developed by Milhes *et al*.^21^. Briefly, genomic DNA of third-stage larvae (500 µL of larval suspension) was extracted using the Power Soil DNA Isolation kit (QIAGEN). All experiments were based on real-time PCR assays using TaqMan technology in simplex PCR reactions. Primers and probes are presented in details in Milhes *et al*.^21^. Standard curves for larval DNA quantitation were established for each PCR run and for the three species *Haemonchus contortus*, *Teladorsagia circumcincta* and *Trichostrongylus colubriformis*. The proportion of one of these species was established by dividing the total number of L3s of this species by the total number of L3s of the three species, both numbers being evaluated from the quantification cycle (Cq) values obtained in Real-Time PCR.

Body condition scores were evaluated by the same person during the whole study and recorded on a 1 to 5 scale. Individual packed cell volumes were obtained with the microhematocrit method (10,000 rpm, 10 minutes at room temperature) immediately after the reception of the EDTA blood samples in the National Veterinary School of Toulouse.

### Statistical analysis

Statistical analyses were performed by the statistical software R Version 3.4.0^22^. The effect of system on EPG, BCS and PCV were analyzed by mixed model including system (cell grazing system versus rotational grazing system), sampling season, age of ewes during sampling, interaction between system and sampling season and that between system and age of ewes during sampling as fixed effects and ewe as random effect. System effects were declared significant at *P* < 0.05, and a trend was assumed for probabilities <0.1 and >0.05. The chi-squared test was carried out to determine the effect of system on proportion of ewes excreting more than 500 EPG and more than 1000 EPG. Pearson’s correlation coefficient was calculated to find out the correlations between EPG, BCS and PCV. For each year, the difference of rest periods between cell grazing and rotational grazing systems was examined by ANOVA (Analysis of Variance). The system effect on EPG of lambs was examined by the Kruskall-Wallis non parametric test.

### Ethics approval

This study was conducted under the agreement N° 2015260-002-ddcspp (Direction Départementale de la Cohésion Sociale et de la Protection des Populations de la Haute Vienne). A regional ethic committee (Région Nouvelle Aquitaine) validated this protocol.

The animals included in this study were sampled by the veterinary practitioner of the farm or under his supervision. Only fecal and blood samples only were collected during the whole study. All treatments of the animals were done by the veterinary practitioner according to previous anthelmintic treatment program. No specific treatment was done for the purpose of the study.

We confirmed that all methods were performed in accordance with relevant guidelines when available. Hematological and coprological analyses were routine analyses. Coprocultures were made according to the guidelines of the MAFF (Ministry of Agriculture, Fisheries and Food). Morphological identifications of GIN infective larvae were performed according to the publication of Van Wyk and Mayhew (2013). Finally, molecular identifications were based on an in-house real-time PCR published in 2017 (Milhes *et al*., 2017) in the journal “Parasitology Research”.

## References

[1] Mavrot F, Hertzberg H, Torgenson P (2015). Effect of gastro-intestinal nematode infection on sheep performance: a systematic review and meta-analysis. Parasites & Vectors.

[2] Keegan J (2017). A nationwide survey of anthelmintic treatment failure on sheep farms in Ireland. Irish Vet. J..

[3] Paraud C (2016). Cross-resistance to moxidectin and ivermectin on a meat sheep farm in France. Vet. Parasitol..

[4] Ploeger H, Everts R (2018). Alarming levels of anthelmintic resistance against gastrointestinal nematodes in sheep in the Netherlands. Vet. Parasitol..

[5] Cazajous T (2018). Multiple-resistance to ivermectin and benzimidazole of a *Haemonchus contortus* population in a sheep flock from mainland France, first report. Vet. Parasitol. Reg. Studies and Reports.

[6] Aguerre S (2018). Resistance to gastrointestinal nematodes in dairy sheep: genetic variability and relevance of artificial infection of nucleus ram to select for resistant ewes on farms. Vet. Parasitol..

[7] Emery DL, Hunt PW, Le Jambre LF (2016). *Haemonchus contortus*: the then and now, and where to from here?. Int. J. Parasitol..

[8] Gaudin E (2016). Efficacy of sainfoin (*Onobrychis viciifolia*) pellets against multi resistant *Haemonchus contortus* and interaction with oral ivermectin: Implications for on-farm control. Vet. Parasitol..

[9] Healey K, Lawlor C, Knox MR, Chambers M, Lamb J (2018). Field evaluation of *Duddingtonia flagrans* IAH 1297 for the reduction of worm burden in grazing animals: Tracer studies in sheep. Vet. Parasitol..

[10] Mahieu M, Aumont G (2009). Effects of sheep and cattle alternate grazing on sheep parasitism and production. Trop. Anim. Health and Prod..

[11] Colvin AF, Walken-Brown SW, Knox MR, Scott JM (2008). Intensive rotational grazing assists control of gastrointestinal nematodosis of sheep in a cool temperate environment with summer-dominant rainfall. Vet. Parasitol..

[12] Walkden-Brown SW (2013). Grazing systems and worm control in sheep: a long-term case study involving three management systems with analysis of factors influencing faecal worm egg count. Anim. Prod. Sci..

[13] Callinan APL, Westcott JM (1986). Vertical distribution of Trichostrongylid larvae on herbage and in soil. Int. J. Parasitol..

[14] O’Connor LJ, Walkden-Brown SW, Kahn LP (2006). Ecology of the free-living stages of major trichostrongylid parasites of sheep. Vet. Parasitol..

[15] Rossanigo CE, Gruner L (1995). Moisture and temperature requirements in faeces for the development of free-living stages of gastrointestinal nematodes of sheep, cattle and deer. J. Helminthol..

[16] Hoste H, Cabaret J (1992). Intergeneric relations between nematodes of the digestive tract in lambs: A multivariate approach. Int. J. Parasitol..

[17] Chylinski C, Lherminé E, Coquille M, Cabaret J (2014). Desiccation tolerance of gastrointestinal nematode third-stage larvae: exploring the effects on survival and fitness. Parasitol. Res..

[18] Raynaud JP (1970). Etude de l’efficacité d’une technique de coproscopie quantitative pour le diagnostic de routine et le contrôle des infestations parasitaires des bovins, ovins, équins et porcins. Ann. Parasitol..

[19] MAFF Ministry of Agriculture, Fisheries and Food Manual of Veterinary Parasitological Laboratory Techniques, Reference book 418 HMSO (1986)

[20] Van Wyk, J. A. & Mayhew, E. Morphological identification of parasitic nematode infective larvae of small ruminants and cattle: A practical lab guide. *Onders*. *J*. *Vet*. *Res*. **80**(1), 10.4102/ojvr.v80i1.539 (2013) 10.4102/ojvr.v80i1.539 23718204

[21] Milhes M (2017). A real-time PCR to identify anthelmintic-resistant nematodes in sheep farms. Parasitol. Res..

[22] R Core Team R: A language and environment for statistical computing. R Foundation for Statistical Computing, Vienna, Austria (2017).

